# Comparative evaluation of temperature generation during laser lithotripsy: thulium fiber laser, pulsed thulium: YAG, and holmium: YAG with pulse modulation in an ex vivo porcine kidney model

**DOI:** 10.1007/s00240-026-01957-8

**Published:** 2026-03-02

**Authors:** Bruce Gao, Tyler Sheetz, Aymon Ali, Luke Griffiths, Harel Sims, Helen Gao, Tiffany R. Huang, Yezan Hadidi, Seyedamirvala Saadat, Jamie Finegan, Vi Nguyen, Sirpi Nackeeran, Seth K. Bechis, Roshan M. Patel

**Affiliations:** 1 Department of Urology, University of California, Irvine, Orange, USA; 2 Department of Urology, University of California, San Diego, San Diego, USA

**Keywords:** Thulium, Holmium, Laser, Temperature, Ureteroscopy

## Abstract

Ureteroscopic laser lithotripsy results in heat generation, raising concern for thermal injury. As laser platforms evolve, comparative assessment of their thermal profiles is increasingly important. This study presents the first comparative evaluation of temperature generation using three contemporary systems: pulsed thulium: YAG (Tm: YAG; Dornier Thulio), thulium fiber laser (TFL; Olympus SOLTIVE), holmium: YAG with pulse modulation (MOSES 2.0; Boston Scientific MOSES 2.0) using manufacturer-recommended dusting settings in an ex vivo porcine kidney model. Eighteen porcine kidney–ureter units were implanted with renal pelvic temperature probes and Bego stones. Specimens were randomized to one of three lithotripsy systems (*n* = 6 per group; one MOSES specimen excluded). A flexible ureteroscope was introduced via a 35 cm 10/12Fr ureteral access sheath, followed by 10 min of near-continuous dusting using 200 μm fibers (0.3 J/50Hz for TFL and Tm: YAG; 0.3 J/60 Hz for MOSES 2.0). Pressurized irrigation was maintained at 100-150mmHg. Outcomes included absolute temperature change, total energy delivered, and temperature increase normalized to energy (°C/kJ). The greatest mean temperature rise was observed with TFL (8.13 ± 1.96 °C), followed by pulsed Tm: YAG (4.12 ± 1.26 °C) and MOSES 2.0 (3.32 ± 1.84 °C). TFL demonstrated significantly greater heat generation than Tm: YAG (*p* = 0.0031) and MOSES 2.0 (*p* = 0.0010). When temperature was adjusted for energy delivery, TFL (1.09 ± 0.30 °C/kJ) surpassed both Tm: YAG (0.47 ± 0.14 °C/kJ; *p* = 0.0007) and MOSES 2.0 (0.31 ± 0.18 °C/kJ; *p* = 0.0001). No specimen exceeded temperatures of 43 °C. In this ex vivo setting, TFL was associated with higher heat generation relative to both pulsed Tm: YAG and MOSES 2.0; however, all systems remained below accepted thermal safety thresholds under the tested conditions (43 °C).

## Introduction

Laser lithotripsy is the dominant energy modality for ureteroscopic management of nephrolithiasis, with continued refinements in laser technology aimed at improving fragmentation efficiency, retropulsion control, and procedural workflow [1]. Despite these advancements, intrarenal heat generation remains an important safety consideration. Laser activation increases the temperature of the surrounding fluid, and experimental and translational studies suggest that temperatures approaching or exceeding 43 °C may result in urothelial or papillary injury [2, 3], particularly during prolonged activation or compromised irrigation conditions. Intrarenal temperature rise depends on multiple factors, including irrigation flow, outflow resistance, laser power settings, activation time, and wavelength-specific water absorption characteristics [4]. Understanding these thermal effects is essential as newer laser platforms are incorporated into clinical practice.

In recent years, several alternative lithotripsy systems have emerged alongside the conventional holmium: YAG (Ho: YAG) laser. The thulium fiber laser (TFL), operating at 1940 nm, delivers high-frequency, low-pulse-energy emission with strong water absorption, producing smooth ablation and reduced retropulsion [5, 6]. Pulsed thulium: YAG (Tm: YAG), at 2013 nm, offers slightly more favorable fluid absorption properties in a solid-state configuration, supporting high-frequency dusting [7]. Meanwhile, pulse-modulated Ho: YAG systems—such as MOSES 2.0—use micro-pulse sequencing to improve energy transmission and cavitational dynamics through a laser generated air-bubble [8]. These differences in wavelength, pulse structure, and temporal energy delivery may influence the magnitude and distribution of heat produced during lithotripsy. However, existing thermal studies vary significantly in methodology, irrigation pressures, access sheath use, and laser settings, limiting direct comparison across platforms [9].

A standardized, controlled evaluation is therefore needed to clarify relative intrarenal temperature effects among these newer systems. To date, no study has directly compared TFL, pulsed Tm: YAG, and MOSES 2.0 in an ex vivo model. Such data are clinically relevant, given increasing adoption of newer laser platforms and the lack of comparative thermal benchmarks under typical ureteroscopic conditions.

The present study provides the first head-to-head ex vivo comparison of thermal generation among pulsed Tm: YAG, TFL, and MOSES 2.0 Ho: YAG systems using factory-recommended dusting settings. By evaluating absolute temperature rise and energy-normalized heat generation, this work offers an objective assessment of intrapelvic temperature profiles across three contemporary laser technologies and informs their safe use during ureteroscopic stone treatment.

## Materials and methods

### Porcine kidney-ureter and stone preparation

Eighteen juvenile female Yorkshire porcine kidney–ureter units were obtained (*Sierra for Medical Science*,* Whittier*,* CA*). Under direct endoscopic visualization, a multipoint temperature-sensing probe (*Galil Medical*,* Arden Hills*,* MN*) was percutaneously introduced into the renal pelvis and secured to the parenchymal surface using a 2 − 0 silk suture to ensure stable positioning throughout the experiment.

Ovoid Bego stones (0.46–1.14 g) were prepared according to established methods and air-dried for 48 h [10]. Each stone was implanted into the renal pelvis through a small pyelotomy, which was subsequently closed in a watertight fashion using a continuous 3 − 0 Vicryl suture.

Kidney–ureter units were then randomly assigned, using a computer-generated randomization sequence, to one of three laser groups: MOSES 2.0, TFL, or pulsed Tm: YAG (*n* = 6 per group).

### Laser lithotripsy and temperature measurements

Kidney–ureter units were submerged in a 37 °C normal saline bath for equilibration. A 35-cm, 10/12 Fr ureteral access sheath (*Cook Medical*,* Bloomington*,* IN*) was advanced such that its distal tip rested at the ureteropelvic junction. An 8.5 F digital flexible ureteroscope (*Karl Storz*,* Tuttlingen*,* Germany*) was then passed through the sheath into the renal pelvis to visualize the implanted stone.

Irrigation was delivered using a bolus-pressurized bag of 0.9% sodium chloride maintained at 150 mmHg. Four fellowship-trained endourologists performed laser dusting of the implanted Bego stone. Near continuous laser lithotripsy was carried out for 10 min using 200-µm laser fibers and factory-recommended dusting settings: 0.3 J/50 Hz for pulsed Tm: YAG and TFL systems, and 0.3 J/60 Hz for the MOSES 2.0 system.

Temperature data were recorded continuously from the temperature probe throughout the entire lithotripsy interval. Temperature values were recorded at 15-second intervals throughout the 10-minute lithotripsy period. Baseline temperature was defined as the intrapelvic temperature recorded immediately prior to laser activation and was obtained directly from the temperature probe. Total laser energy was acquired from the laser platform at the conclusion of each session.

**Fig. 1:**
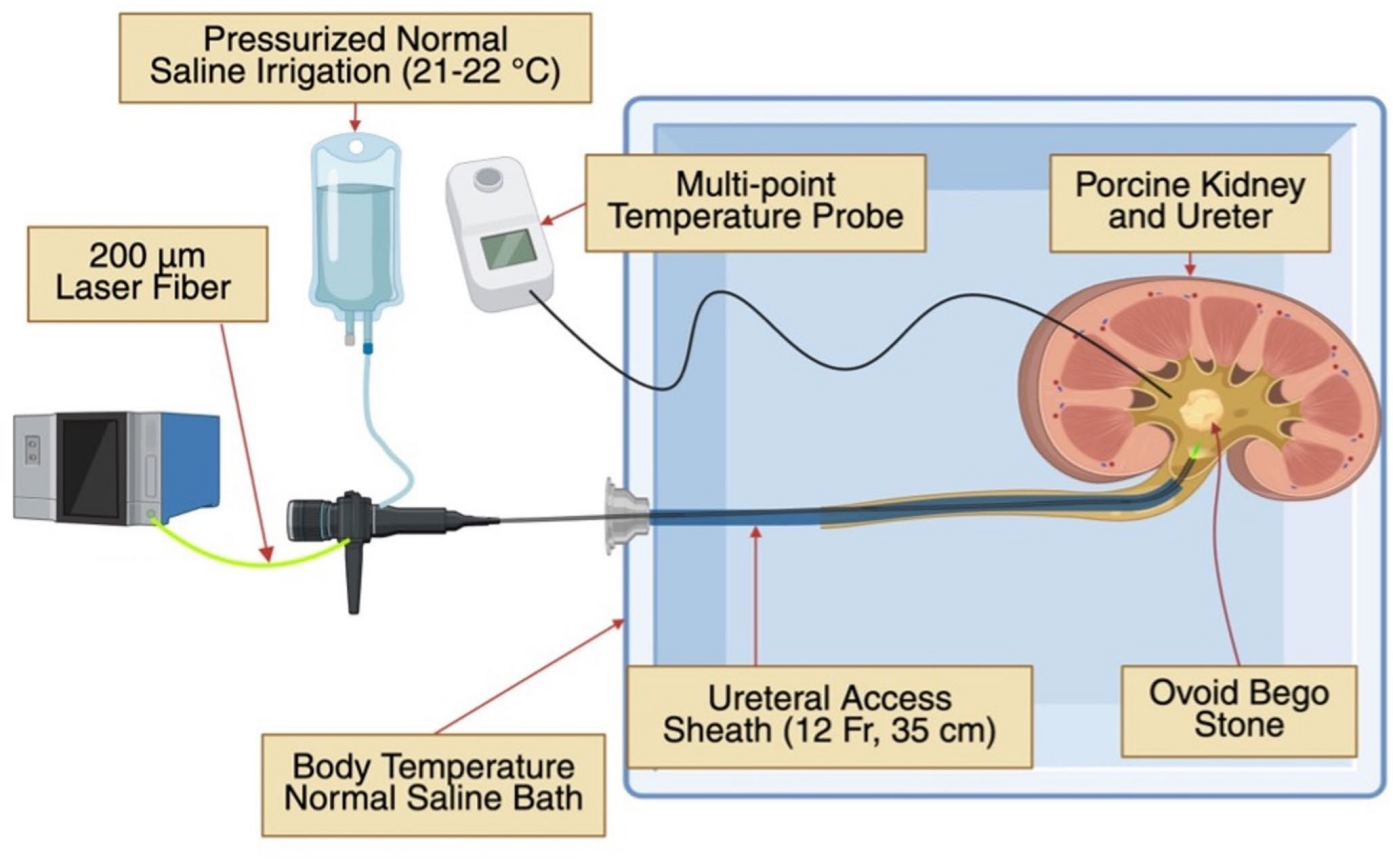
Ex vivo porcine model showing renal pelvic temperature probe placement, Bego stone implantation, and ureteroscopic laser lithotripsy performed through a 12 Fr access sheath under pressurized irrigation. Created in BioRender. Gao, B. (2025) https://BioRender.com/zpv5bce

A schematic representation of the completed ex vivo laser lithotripsy set-up is shown in Fig. 1.

### Statistical analysis

For each renal unit, maximum intrapelvic temperature change was calculated as the difference between the highest temperature recorded during the lithotripsy interval and the baseline temperature. Temperature values exceeding two standard deviations from the group mean were classified as outliers and excluded from primary temperature-change calculations to account for situations such as inadvertent laser contact with the temperature probe or irrigation bag changes. Mean temperature change (°C), total delivered laser energy (kJ), and energy-normalized temperature change (°C/kJ) were calculated for each laser group.

Data normality was assessed using the Shapiro–Wilk test. Comparisons among the three laser systems were performed using one-way analysis of variance (ANOVA) with Tukey’s post hoc testing for pairwise differences. All statistical analyses were conducted using GraphPad Prism 10.1 for macOS (GraphPad Software, Boston, MA). Biostatistical guidance was provided by the Chao Family Comprehensive Cancer Center Biostatistics Shared Resource at the University of California, Irvine.

## Results

### Overview

A total of 18 ex vivo porcine kidney–ureter units underwent 10 minutes of laser lithotripsy (Table 1). One specimen in the MOSES 2.0 group was excluded due to temperature probe malfunction, resulting in final group sizes of MOSES 2.0 (*n* = 5), pulsed thulium: YAG (*n* = 6), and TFL (*n* = 6).

**Table 1:** Mean temperature change and laser energy applied during 10 min of continuous laser lithotripsy in an ex vivo porcine renal pelvis for pulsed Tm: YAG, TFL, and MOSES 2.0 at dusting settings

Laser	MOSES 2.0 (*n* = 5)	Pulsed Tm: YAG (*n* = 6)	TFL (*n* = 6)	*p*-value
Mean temperature change, °C (SD)	3.32 (1.84)	4.12 (1.26)	8.13 (1.96)	0.0006
Mean laser energy, kJ (SD)	10.60 (0.18)	8.73 (0.15)	7.73 (1.76)	0.0016
Mean temperature change per kilojoule °C/kJ (SD)	0.31 (0.18)	0.47 (0.14)	1.09 (0.30)	< 0.0001
Temperature > 43 °C	No	No	No	NA

### Mean temperature change

**Fig. 2:**
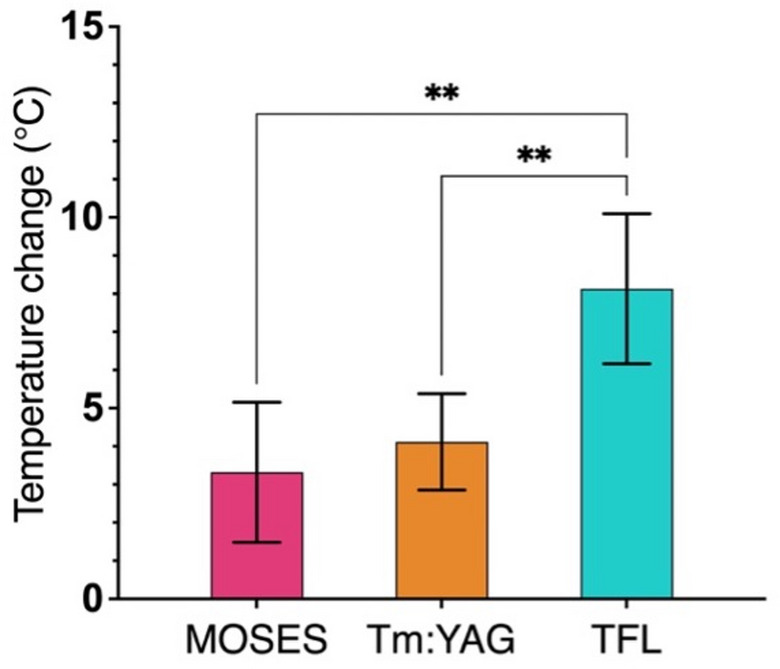
Mean temperature changes during 10 min of continuous laser lithotripsy in an ex vivo porcine renal pelvis with holmium: YAG with pulse modulation (MOSES; Boston Scientific MOSES 2.0), pulsed thulium: YAG (Tm: YAG; Dornier Thulio), and thulium fiber laser (TFL; Olympus SOLTIVE) set to dusting parameters of 0.3 J and 50–60 Hz (*n* = 6). * indicates *p* < 0.05; ** indicates *p* < 0.01; *** indicates *p* < 0.001

The highest mean temperature change observed during laser lithotripsy occurred with TFL, reaching 8.13 ± 1.96 °C. Pulsed Tm: YAG generated a mean temperature change of 4.12 ± 1.26 °C, while MOSES resulted in the lowest mean temperature change at 3.32 ± 1.84 °C. There was no statistically significant difference between MOSES and pulsed Tm: YAG (*p* = 0.7266); however, TFL showed a significantly greater temperature increase compared to both pulsed Tm: YAG (*p* = 0.0031) and MOSES (*p* = 0.0010) (Fig. 2). There were no episodes where irrigation fluid temperature exceeded 43 °C within all three lasers.

### Mean temperature change per kilojoule

**Fig. 3:**
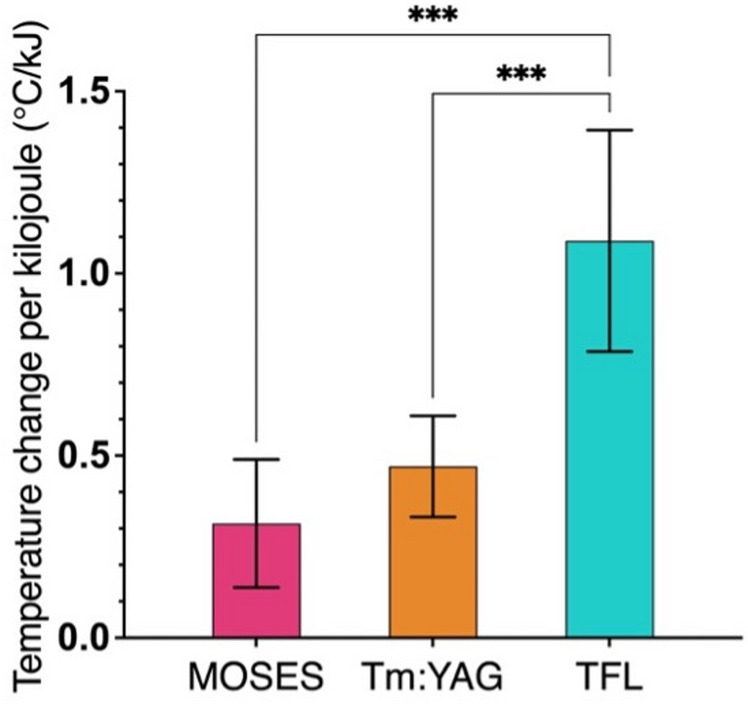
Mean temperature change per kilojoule during 10 min of continuous laser lithotripsy in an ex vivo porcine renal pelvis with holmium: YAG with pulse modulation (MOSES; Boston Scientific MOSES 2.0), pulsed thulium: YAG (Tm: YAG; Dornier Thulio), and thulium fiber laser (TFL; Olympus SOLTIVE) set to dusting parameters of 0.3 J and 50–60 Hz (*n* = 6). * indicates *p* < 0.05; ** indicates *p* < 0.01; *** indicates *p* < 0.001

Mean total laser energy delivered was highest for MOSES 2.0 (10.60 ± 0.18 kJ), followed by Tm: YAG (8.73 ± 0.15 kJ) and TFL (7.73 ± 1.76 kJ). Pairwise comparisons revealed significant differences between MOSES and Tm: YAG (*p* = 0.0218) and between MOSES and TFL (*p* = 0.0111), while no difference was observed between Tm: YAG and TFL (*p* > 0.9999). When normalized for energy, TFL produced a significantly greater temperature rise per kilojoule (1.09 ± 0.30 °C/kJ) compared with both Tm: YAG (0.47 ± 0.14 °C/kJ; *p* = 0.0007) and MOSES 2.0 (0.31 ± 0.18 °C/kJ; *p* = 0.0001). There was no difference between MOSES 2.0 and Tm: YAG (*p* = 0.4899) (Fig. 3).

## Discussion

This study offers the first head-to-head comparison of intrapelvic temperature profiles generated by pulsed thulium: YAG (Tm: YAG), thulium fiber laser (TFL), and holmium: YAG with pulse modulation (MOSES 2.0) under standardized dusting parameters in an ex vivo porcine kidney model. The principal finding was that TFL produced significantly higher intrapelvic temperature elevations than both Tm: YAG and MOSES 2.0, despite using less total energy. Nevertheless, under conditions of active irrigation and unobstructed outflow using a 10/12 Fr ureteral access sheath above the ureteropelvic junction at 150 mmHg, none of the systems exceeded the commonly cited 43 °C thermal safety threshold [2–4] during 10 min of near-continuous activation. These findings suggest that, in settings with sufficient irrigation and drainage, all three laser platforms are capable of operating within a thermally safe range.

The greater temperature elevations observed with TFL are consistent with its emission profile and the higher water absorption coefficient at its 1940 nm wavelength [5]. Although these properties enhance vaporization efficiency, they also confine thermal deposition to a smaller intraluminal volume. By contrast, MOSES 2.0 produced the lowest temperature rise, aligning with its dual-pulse modulation intended to optimize cavitational energy transfer while limiting residual heat with a lower water absorption coefficient (2140 nm) [4]. Pulsed Tm: YAG demonstrated intermediate thermal behavior, reflecting its middle fluid absorption wavelength (2013 nm) between the TFL and holmium systems. Energy-normalized comparisons further highlight these mechanistic distinctions: on a per-kilojoule basis, TFL generated more than twice the temperature increase observed with either MOSES 2.0 or Tm: YAG. Thermal dose metrics such as cumulative equivalent minutes at 43 °C (CEM43) provide additional biological context beyond peak temperature alone. In this study, no specimen exceeded 43 °C during lithotripsy, and under these subthreshold conditions any calculated CEM43 would be extremely low and well below injury-associated thresholds. Given the use of room-temperature irrigation, which likely provided convective cooling and conservative temperature estimates, analysis focused on peak temperature change and energy-normalized heat generation, consistent with the format of prior endourologic thermal studies.

Several prior experimental studies have reported minimal or no clinically meaningful differences in intrarenal heat generation among Ho: YAG and thulium-based laser systems, and these findings should be interpreted in the context of the experimental conditions under which they were obtained. For example, Okhunov et al. demonstrated in an in vivo porcine model that TFL did not result in sustained suprathreshold intrarenal temperatures; in that study, a 12/14 Fr ureteral access sheath was used, and physiologic renal perfusion in the live animal likely provided additional convective cooling [11]. Similarly, Jiang et al. reported comparable intrarenal temperature profiles between TFL and Ho: YAG in an in vivo porcine model, with no temperatures exceeding 44 °C, again using a larger 12/14 Fr access sheath and benefiting from blood-flow–mediated heat dissipation [12]. Accordingly, the present findings should be viewed as complementary to the existing literature, reflecting relative thermal behavior under the specific experimental conditions and setup employed (10/12 Fr ureteral access sheath advanced to the ureteropelvic junction in an ex vivo environment).

The experimental model incorporated a 10/12 Fr ureteral access sheath advanced into the renal pelvis past the ureteropelvic junction and continuous pressurized irrigation (150 mmHg), both of which promote robust inflow and outflow. Accordingly, these findings may not fully translate to scenarios lacking an access sheath, to cases in which the sheath terminates below the ureteropelvic junction, or to situations with reduced irrigation. Localized environments with restricted egress—such as a calyx with a tight infundibulum. a narrow ureter, or sheath below the ureteropelvic junction—may exhibit substantially different thermal behavior. This concern is supported by an in vivo porcine study demonstrating that TFL activation without a ureteral access sheath can lead to intrarenal temperatures exceeding 44 °C under similar dusting and fragmentation settings [11]. Additionally, even the holmium laser without a ureteral access sheath has been demonstrated to generate high intrarenal temperatures (43.1 °C) in an ex vivo porcine kidney model. Thus, while all systems operated within safe thermal limits under the conditions tested, the results should be interpreted within the context of the model with ample fluid exchange (150 mmHg irrigation pressure with a 10/12 F UAS above the ureteropelvic junction). However, all this considered, the data are likely generalizable to settings with larger-caliber access sheaths (14–16 Fr) or a 10/12 Fr sheath used with higher irrigation pressures, where flow dynamics would be expected to be equal or more favorable than in the present study [12].

This study has several limitations. It was conducted ex vivo, without physiologic perfusion or urine production to provide additional convective cooling. Temperature measurements were limited to the renal pelvis and therefore may not reflect microenvironmental temperatures at the laser tip or within anatomically constrained spaces. Furthermore, heat conducted directly into the stone was not assessed; consequently, while bulk fluid temperatures remained within safe limits, the peak temperature achieved within the stone itself cannot be inferred. Despite these constraints, the use of standardized methodology, similar laser parameters, and a setup that closely approximates practical operating conditions permits a valid ex vivo comparison of relative thermal behavior among the evaluated systems for the first time in literature.

Furthermore, laser parameters were selected using manufacturer-recommended dusting settings; however, the MOSES 2.0 system has a minimum allowable pulse frequency of 60 Hz at 0.3 J, which precluded exact matching to the 50 Hz settings used for the thulium-based systems at the same pulse energy. As a result, a small difference in nominal power (15–18 W) was unavoidable. To mitigate this limitation, temperature change was additionally normalized to the exact total energy delivered, yielding consistent relative differences across platforms. Nonetheless, manufacturer-recommended settings are not necessarily optimized for thermal efficiency or safety, and future studies using fully matched power and duty-cycle conditions may further refine comparative thermal assessments. Finally, laser activation duration and firing pattern represent important determinants of intrarenal heat generation. In this study, near-continuous laser activation over a 10-minute interval was used to approximate typical clinical lithotripsy behavior rather than a fixed-duty-cycle paradigm; however, this approach may introduce inter-operator variability, particularly in a bench model involving multiple surgeons. Accordingly, this represents a limitation of the study, and future investigations incorporating both strictly controlled activation protocols and clinically simulated lithotripsy conditions may further refine comparative thermal assessments.

## Conclusion

In this ex vivo porcine model, TFL was associated with greater mean and energy-normalized intrapelvic temperature increases compared with pulsed thulium: YAG and holmium: YAG with pulse modulation. However, under the specific experimental conditions studied—use of a 10/12 Fr ureteral access sheath positioned past the ureteropelvic junction and continuous room temperature irrigation at 150 mmHg—no laser system exceeded the commonly cited 43 °C safety threshold during 10 minutes of dusting.

## Data Availability

The data generated within this study are available upon request.

## Abbreviations

ANOVA: Analysis of Variance
Bego: (Brand name; artificial stone material used in studies)
°C: Degrees Celsius
°C/kJ: Degrees Celsius per kilojoule
Fr: French (catheter size)
F: French (scope size, as in 8.5 F)
Hz: Hertz
Ho:YAG: Holmium:Yttrium Aluminum Garnet
J: Joule
kJ: Kilojoule
mmHg: Millimeters of mercury (pressure)
MOSES 2.0: (Proper name; Boston Scientific pulse-modulated Ho:YAG system)
nm: Nanometer
PEARLS: Prospective Evaluation of stone composition using Attenuation-based Radiographic and Laser Stratification (from cited study)
SD: Standard Deviation
TFL: Thulium Fiber Laser
Tm:YAG: Thulium:Yttrium Aluminum Garnet
UAS: Ureteral Access Sheath
UPJ: Ureteropelvic Junction
VS: Versus (comparative studies)

## References

[1] Kronenberg P, Somani B (2018) Advances in Lasers for the Treatment of Stones - A Systematic Review. Curr Urol Rep 19:45. 10.1007/s11934-018-0807-y 29774438 10.1007/s11934-018-0807-y PMC5958148

[2] Peteinaris A, Tsaturyan A, Bravou V et al (2023) High-Power Laser Lithotripsy - Do We Treat or Harm? Histological Evaluation of Temperature Effects in an In Vivo Study with Thulium Fiber Laser. Cent Eur J Urol 76:44–48. 10.5173/ceju.2023.24 10.5173/ceju.2023.24 PMC10091897 37064255

[3] Sapareto SA, Dewey WC (1984) Thermal Dose Determination in Cancer Therapy. Int J Radiat Oncol Biol Phys 10:787–800. 10.1016/0360-3016(84)90379-1 6547421 10.1016/0360-3016(84)90379-1

[4] Multescu R, Geavlete P, Georgescu D et al (2025) Temperature Increase During Flexible Ureteroscopic Approach with Holmium Laser Lithotripsy: How Much Should We Be Concerned? Med (Kaunas) 61. 10.3390/medicina61081335 10.3390/medicina61081335 PMC12387338 40870380

[5] Gao B, Bobrowski A, Lee J (2021) A Scoping Review of the Clinical Efficacy and Safety of the Novel Thulium Fiber Laser: The Rising Star of Laser Lithotripsy. Can Urol Assoc J 15:56–66. 10.5489/cuaj.6804 32744995 10.5489/cuaj.6804 PMC7864720

[6] Cumpanas AD, Katta N, Vu TN et al (2025) Warm irrigation fluid effect on Thulium fiber laser (TFL) ablation of uroliths. Lasers Med Sci 40:112. 10.1007/s10103-024-04253-2 39982499 10.1007/s10103-024-04253-2

[7] Kwok J-L, Ventimiglia E, De Coninck V et al (2023) Pulsed Thulium:YAG Laser - Ready to Dust All Urinary Stone Composition Types? Results from a PEARLS Analysis. World J Urol 41:2823–2831. 10.1007/s00345-023-04549-y 37587366 10.1007/s00345-023-04549-y PMC10581948

[8] Martinez B, Ntasiotis P, Katsakiori P et al (2024) Assessment of Stone Ablation Rate Using the Moses Technology Modes with Different Energy and Pulse Settings: An Experimental Study. Arab J Urol 22:131–137. 10.1080/20905998.2023.2301641 38818253 10.1080/20905998.2023.2301641 PMC11136463

[9] Kim HJ, Hong SK (2025) Rise in Intraluminal Temperature During Ureteroscopy: Is This A Concern? Investig Clin Urol 66:1–10. 10.4111/icu.20240369 39791579 10.4111/icu.20240369 PMC11729224

[10] Esch E, Simmons WN, Sankin G et al (2010) A Simple Method For Fabricating Artificial Kidney Stones of Different Physical Properties. Urol Res 38:315–319. 10.1007/s00240-010-0298-x 20652562 10.1007/s00240-010-0298-x PMC3752343

[11] Okhunov Z, Jiang P, Afyouni AS et al (2021) Caveat Emptor: The Heat Is ON-An In Vivo Evaluation of the Thulium Fiber Laser and Temperature Changes in the Porcine Kidney During Dusting and Fragmentation Modes. J Endourol 35:1716–1722. 10.1089/end.2021.0206 33906433 10.1089/end.2021.0206

[12] Jiang P, Okhunov Z, Afyouni AS et al (2023) Comparison of Superpulse Thulium Fiber Laser vs Holmium Laser for Ablation of Renal Calculi in an In Vivo Porcine Model. J Endourol 37:335–340. 10.1089/end.2022.0445 36401505 10.1089/end.2022.0445

